# Longevity of resin-bonded fixed partial dental prostheses made with metal alloys

**DOI:** 10.1007/s00784-015-1619-9

**Published:** 2015-10-06

**Authors:** Naomi Tanoue

**Affiliations:** Department of Pediatric Dentistry, Nagasaki University Hospital, 1-7-1 Sakamoto, Nagasaki, 852-8501 Japan

**Keywords:** Resin adhesive, Debonding, Resin-bonded fixed partial dental prosthesis, Survival

## Abstract

**Objectives:**

The purpose of this study was to evaluate the clinical performance of resin-bonded fixed partial dental prostheses (RBFPDPs) made with metal alloys.

**Materials and methods:**

The retention of 311 RBFPDPs from 226 patients fabricated from 1983 to 2013 using an adhesive resin was clinically evaluated. Partial or complete debonding of the RBFPDP or framework fracture was considered a treatment failure. All data were obtained from clinical examinations, and missing data were censored at the date of the last available information. The effect of the following factors on survival rate were investigated: patient gender, location (maxilla/mandible and anterior/posterior), number of missing teeth, number of abutment teeth, framework structure, type of metal alloy, patient age at the point of cementation, cement type, and distinction of the treating dentist. Data were analyzed with the Kaplan–Meier survival tests, log-rank tests, and Cox regression analyses (*α* = 0.05).

**Results:**

The Kaplan–Meier survival rate was 41.2 % ± 6.5 % (standard error) at 28.8 years (last outcome event). Significant differences were found for patient age and treating dentist (*p* < 0.05). The risk of failure in younger patients was 1.7 times greater than that in older patients and that of inexperienced dentists was 2.0 times greater than that of dentist experienced and specialized in adhesive dentistry.

**Conclusions:**

When fabricating RBFPDPs for younger patients, mechanical preparation for bonding may be necessary in consideration of the risk for debonding. Experienced dentists may achieve better results.

**Clinical relevance:**

Mastery of skills is necessary to ensure excellent prognoses for RBFPDPs.

## Introduction

The resin-bonded fixed partial dental prosthesis (RBFPDP) is a conservative method for replacing missing teeth. To conserve tooth structure, the minimal preparation required for RBFPDPs is clinically advantageous.

Because the adhesive system for base metals was established in the early 1980s, many dentists have treated intermediary missing teeth using the RBFPDP method in dental clinical practice, and clinical evaluations of RBFPDPs have also been performed [1–6]. In particular, after the establishment of the noble metal adhesion system in the late 1980s, more RBFPDPs using noble and/or base metal alloys have been performed [7–11]. However, Dunne and Millar reported a high failure rate of RBFPDPs in comparison with conventional fixed partial dentures (FPDs) [12].

The longevity or prognosis of RBFPDPs are thought to be influenced by various factors such as preparation, type of metal alloy, treatment of the adhesive surface, type of cement, number of abutment teeth, number of missing teeth, location of the prosthesis, dentition, patient age, operator skill, and periodontal disease risk of the patient. The RBFPDP should be designed to avoid risk factors as much as possible, and many researchers have performed investigations to identify these factors. Nevertheless, factors influencing the survival rates of RBFPDPs vary according to the report. For example, many previous reports have indicated statistically higher survival rates of RBFPDPs set in the maxilla than the mandible [2, 13–17], but many recent reports have shown that the position of RBFPDPs (maxilla or mandible) may be unrelated to the survival rate [9, 11, 18–21]. Additionally, the reported effects of other factors on the prognosis have varied. The etiology as to the prognosis and success rates of RBFPDPs remains unclear.

The Prosthodontic Division of Nagasaki University Hospital is the organization that has eagerly carried out basic and clinical research on RBFPDPs. The dentists belonging to the organization inserted many RBFPDPs after the first RBFPDP was inserted in 1983. Thereafter, many cases considered unsuitable for conventional FPDs or implants, but suitable for RBFPDPs, were treated in the Prosthodontic Division. A clinical study with sufficient cases is paramount to obtain reliable evidence. This prospective cohort study performed in the Nagasaki University Hospital is thought to be useful for evaluating the clinical progress of RBFPDPs.

The purpose of the present study was to collect survival data for RBFPDPs made from metal alloys inserted under controlled clinical conditions and to investigate the factors influencing survival rate by evaluating the status of the frameworks.

## Materials and methods

The clinical protocol was approved by the Ethical Committee for Clinical Practice of the Nagasaki University Graduate School of Biomedical Sciences (Approval No. 23).

The RBFPDP in this study was defined as a fixed partial dental prosthesis made from a metal alloy having at least one resin-bonded retainer prepared using the concept of minimal intervention. The insertion period lasted from 1983 to 2013, and inclusion criteria comprised only the need for the RBFPDP to be retained on at least one intact (or exhibiting only minimal lesions that would not interfere with bonding) abutment tooth. However, cantilevered RBFPDPs were excluded.

Three hundred and twenty-five patients visited the Nagasaki University Hospital of Dentistry during the evaluation period, and 429 RBFPDPs met the inclusion criteria. All consecutive patients were asked to participate in this research and undergo recall two to four times per year, depending on the risk factors related to their dentition. The majority agreed to participate, but a significant number of patients who visited the hospital only for RBFPDP treatment preferred to be recalled at their initial dental clinic. Consequently, 99 patients refused participation in this research or in the recall program for personal or no specific reason, and 311 RBFPDPs from 226 patients were evaluated in this study. All participants signed informed consent forms. The distribution of participant age when the RBFPDPs were seated is shown in Table 1. The data were collected by one dentist to a personal computer with high security.

**Table 1:** Distribution of participant age when resin-bonded fixed partial dental prosthesis was fitted

Age	<29	30–39	40–49	50–59	60–69	70–79	>80	Total
*N*	26	22	50	94	82	35	2	311
Percent	8.4	7.1	16.1	30.2	26.4	11.3	0.6	100

All treatments were administered by 18 dentists. The inclusion criterion for the practitioners was to have been working for the Department of Fixed Prosthodontics (at the time) in Nagasaki University Hospital and to be clinically experienced. All RBFPDPs were seated at Nagasaki University Hospital.

**Fig. 1:**
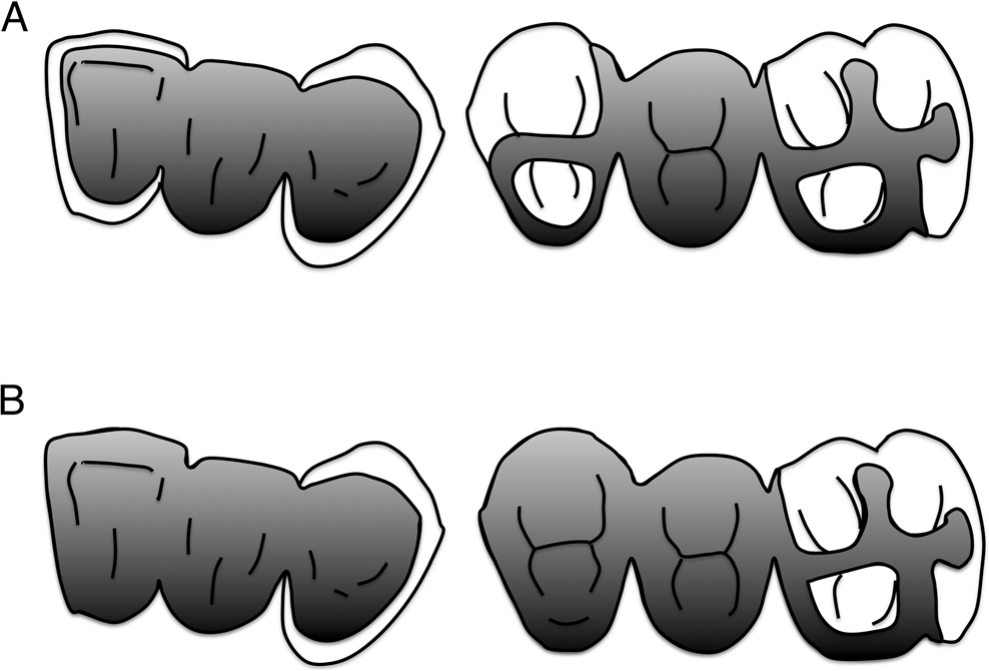
Typical designs for **a** surface-retained and **b** combination of RBFPDPs

The basic retainer design for anterior teeth included a surface bonding wing without a deep groove, according to the method of a previous report [18], and that for posterior teeth included a groove, plate, and strut (GPS) retainer [22]. The typical designs for anterior and posterior RBFPDPs are presented in Fig. 1. Both preparations had supragingival finishing lines. In this study, RBFPDPs where at least one abutment tooth comprised a retainer design were included as subjects. In the case of endodontically treated teeth, a full coverage crown or veneered crown was adopted after adequate core foundation restoration. The RBFPDP frameworks were therefore categorized into two groups: RBFPDPs retained with a wing or GPS retainer (surface-retained) and RBFPDPs with a combination of wing or GPS retainer and full coverage crown (combination).

The frameworks of the RBFPDPs were made of a silver-palladium-copper-gold alloy (Castwell M.C. 12; G-C Corp., Tokyo, Japan), a cobalt-chromium alloy (Biocast; High-Dental Japan, Osaka, Japan), a type 4 gold alloy (Casting Gold M.C.; G-C Corp.), or a multipurpose gold casting alloy (Pontol LFC; Metalor, Neuchatel, Switzerland). The Castwell alloy comprised an age-hardened silver-based casting alloy consisting of 46 % Ag, 20 % Cu, 20 % Pd, and 12 % Au. The Biocast alloy consisted of 64 % Co, 25 % Cr, 5 % Mo, and others; the Casting Gold M.C. alloy consisted of 70 % Au, 14 % Cu, 4 % Au, 3 % Pt, and 3 % Pd; and the Pontol LFC alloy consisted of 70 % Au, 12 % Ag, 9 % Pt, and 6 % Cu. According to the manufacturers, the Castwell and Casting Gold M.C. alloys cannot be fused to porcelain, while Biocast and Pontol LFC alloys can be. The Biocast alloy was mainly used before the release of a noble metal primer in the 1990s. The choice of alloy type was entrusted to the operator. In this study, all alloys except the Co-Cr alloy were classified as noble metals.

The RBFPDPs were fabricated after heavy-body/light-body impressions were taken and maximal intercuspal position was registered using a vinyl polysiloxane impression material. Almost all pontics were veneered for aesthetic purposes either with indirect composite in the case of Castwell M.C. 12 and Casting Gold M.C., or with porcelain in the case of Biocast or Pontol LFC. The pontics were fabricated to contact only in maximal intercuspal position.

All inner surfaces, including those of the full coverage crowns, were airborne-particle abraded for 15 s with 50–70 μm alumina (Hi-aluminas; Shofu Inc., Kyoto, Japan) using an airborne-particle abrader (CL-FSG94; Heraeus Kulzer Inc., Armonk, NY, USA). Regarding the Biocast alloy, the bonding surface was unprimed or treated with primer consisting of an acidic functional monomer for base metal alloys. The surfaces of the other three metal alloys were treated with thione primers for noble metal alloys.

The bonding surface of the abutment tooth was polished with a brush and fluoride-free pumice, and etched in accordance with the manufacturer’s instructions for the cement being used. All RBFPDPs were seated using adhesive resin cements, such as a methyl methacrylate (MMA)-based self-curing resin (Super-Bond C&B; Sun Medical Co. Ltd., Moriyama, Japan) and a composite luting agent (Panavia EX, Panavia 21 or Panavia F2.0; Kuraray Co. Ltd., Osaka, Japan). The full coverage crowns were luted with glass ionomer or resin-modified glass ionomer cements. Restorations were inserted under relatively dry conditions using cotton rolls; a rubber dam was not always applied. All patients were given oral hygiene instructions with special emphasis on cleaning proximal surfaces with an interdental brush and dental floss, and recalled for oral hygiene and RBFPDP evaluations. The baseline for assessment of survival was the date of insertion of the prosthesis.

In this study, the parameters characterized as failure were (1) partial or complete debonding of the framework and (2) fracture of the framework. The attending doctor evaluated the status of the RBFPDP by clinical examination to avoid overlooking a RBFPDP failure, and listed this in the patient’s medical record. Framework debonding was determined by probing the interface between the retainer casting and the tooth with an explorer tip. Restorations that exhibited complete debonding but no major defects were rebonded but judged as a failure, because the survival rate of rebonded RBFPDPs has previously been reported as unacceptable [2]. Other problems, such as abutment tooth caries unrelated to the RBFPDP and fracture of tooth structure without debonding of the RBFPDP were not considered as failures but as end-points. Missing data were censored at the date of the last available information. Radiographs were not systematically taken.

As a result, the 10 variables analyzed were (1) patient gender, (2) location of the RBFPDP (maxilla versus mandible), (3) location of the RBFPDP (anterior versus posterior), (4) number of missing teeth (1 versus >1), (5) number of abutment teeth (2 versus >2), (6) framework structure (surface-retained versus combination), (7) framework alloy (noble metal alloys versus Co-Cr alloy), (8) age of the patient at insertion (≤56 versus >56), (9) cement type (MMA- versus composite-based), and (10) different operators (Dr. A versus Dr. B versus others). The location of the RBFPDP as in (3) refers to the location of the missing tooth/teeth. Regarding (8), the data were divided into young (≤56) and aged (>56) groups at the median age of 56. For (10), the RBFPDP was classified into three groups according to the treating practitioner: Dr. A, Dr. B, and others. Drs. A and B treated the most RBFPDPs. Dr. A commenced RBFPDP treatment 15 years post-graduation and had been inserting RBFPDPs for 25 years, while Dr. B had been placing RBFPDPs for 25 years, commencing immediately after graduation. The remaining 16 dentists were classified as “others,” because their treatment experience and ages varied and the number of cases treated by each was not as substantial.

The survival distributions for each variable were compared using the Kaplan–Meier survival analysis and the Mantel–Cox log-rank test (*α* = 0.05). After drawing the Kaplan–Meier survival curves, the factors expected to affect survival rate were chosen. The effect of each selected variable on survival characteristics was analyzed using the Cox proportional hazards regression model (final model). The effect was expressed as the hazard ratio with 95 % confidence intervals of a particular category compared with the reference category. For all statistical analyses, JMP 10 software (SAS Institute Japan, Tokyo, Japan) was used.

## Results

Two hundred and twenty-nine patients who participated in this study were recalled at least once for examination. The shortest evaluation period was 4 months (one examination after insertion, for health reasons).

**Fig. 2:**
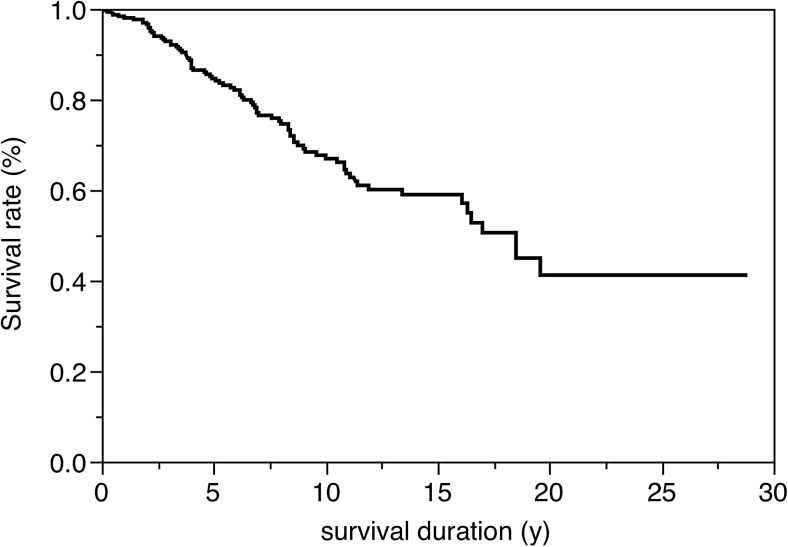
Kaplan–Meier survival curve for all RBFPDPs

Figure 2 shows the Kaplan–Meier survival curve for all RBFPDPs. The maximum observation period was 28.8 years, and the mean observation time was 13.9 years. The corresponding survival ratio of the maximum observation duration was 41.2 %. Among the 311 prostheses assessed, 84 RBFPDPs were evaluated as failures. Six RBFPDP frameworks had fractured; five were made from silver-palladium-copper-gold alloy, and the remaining one was fabricated from Co-Cr alloy. The failures of the remaining 78 RBFPDPs indicated partial or complete debonding, and 13 of the RBFPDPs with complete debonding could be rebonded because the abutment teeth exhibited no defects or aesthetic problems and periodontal support was sufficient. In the 13 RBFPDPs rebonded, eight were made from silver-palladium-copper-gold alloy and the remaining five were fabricated from Co-Cr alloy. Some RBFPDPs that completely detached could not be rebonded because of secondary caries and/or metal deformation. Regarding the 71 RBFPDPs that could not be rebonded, the missing tooth/teeth were treated with new RBFPDPs or other prosthetic methods such as implants and removable prostheses. All failures in the combination design were attributed to debonding of the retainer, and there were no crown failures.

**Fig. 3:**
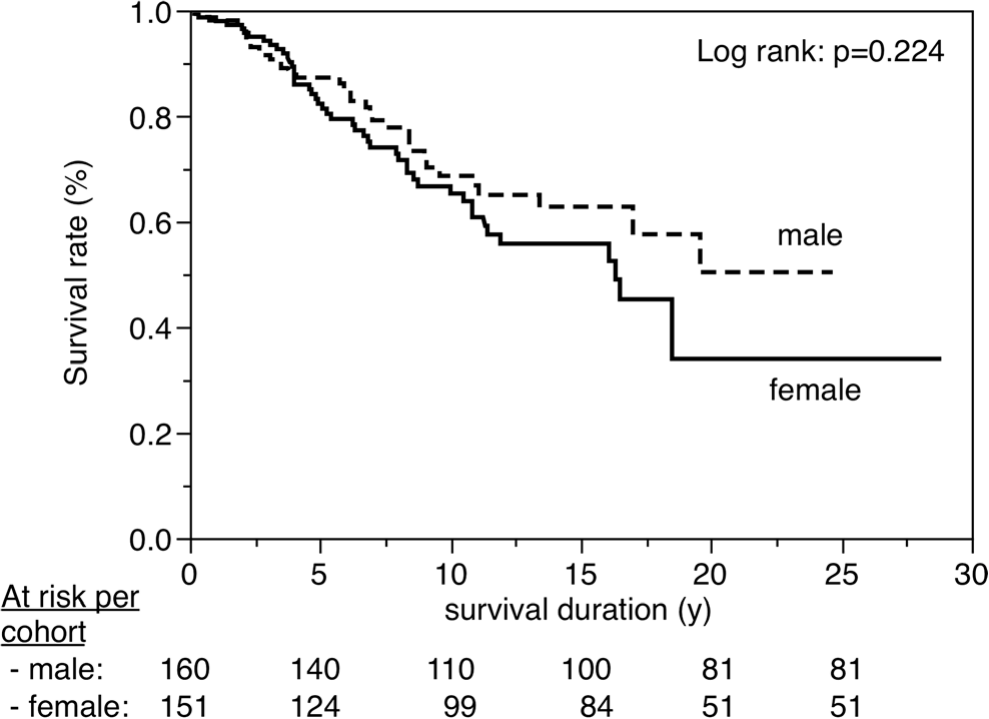
Survival in relation to patient gender (male versus female)

**Fig. 4:**
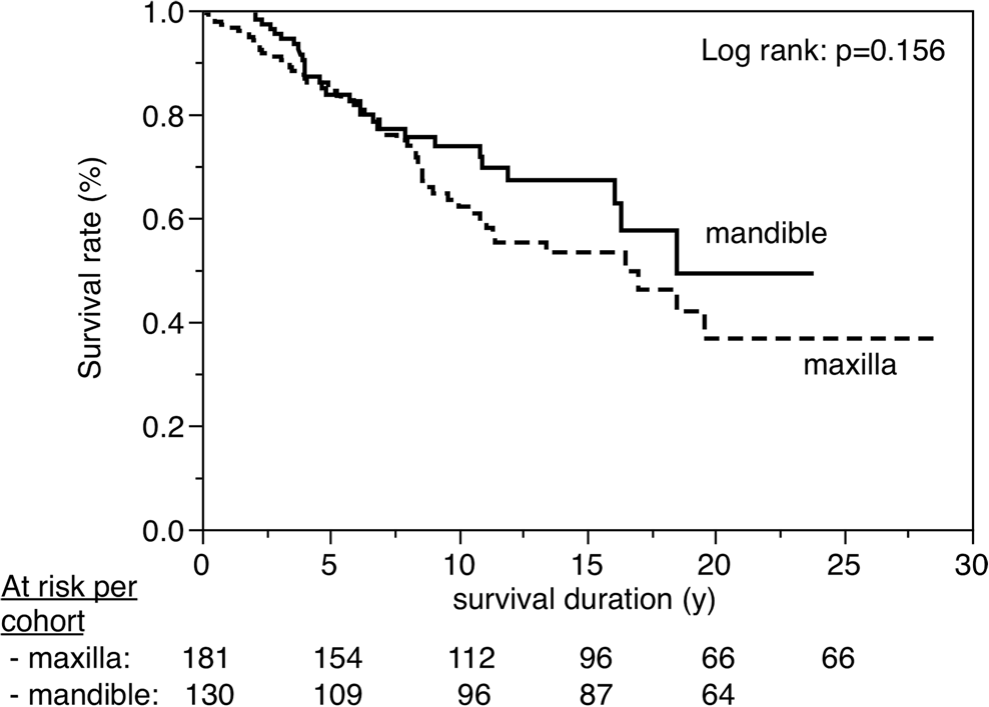
Survival in relation to location of the RBFPDP (maxilla versus mandible)

**Fig. 5:**
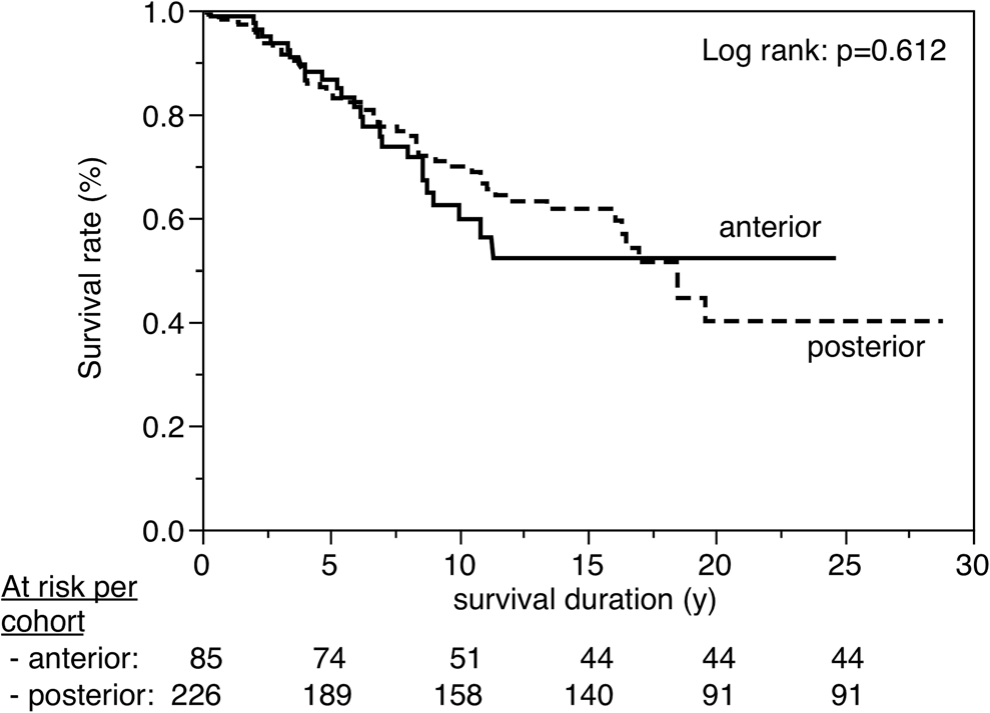
Survival in relation to location of the RBFPDP (anterior versus posterior)

**Fig. 6:**
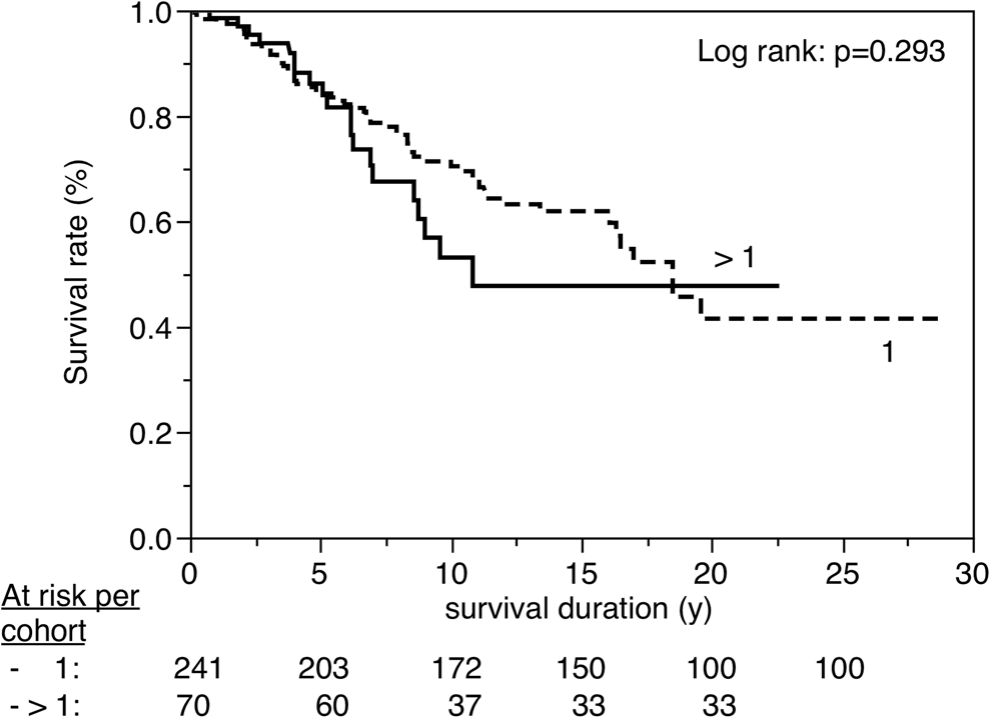
Survival in relation to the number of missing teeth (1 versus >1)

**Fig. 7:**
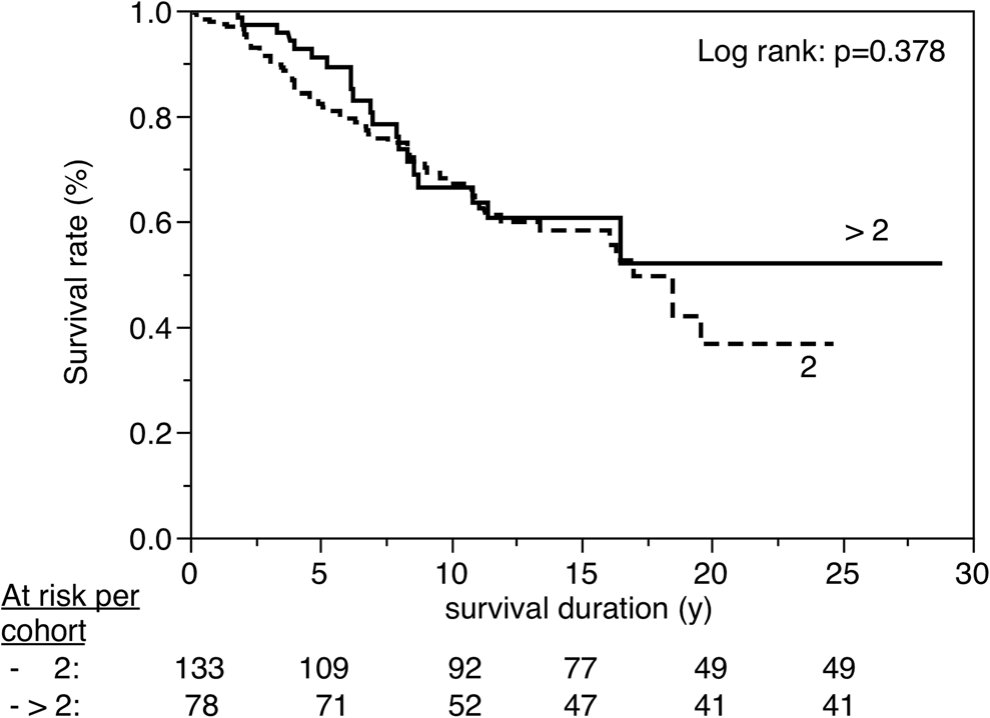
Survival in relation to the number of abutment teeth (2 versus >2)

**Fig. 8:**
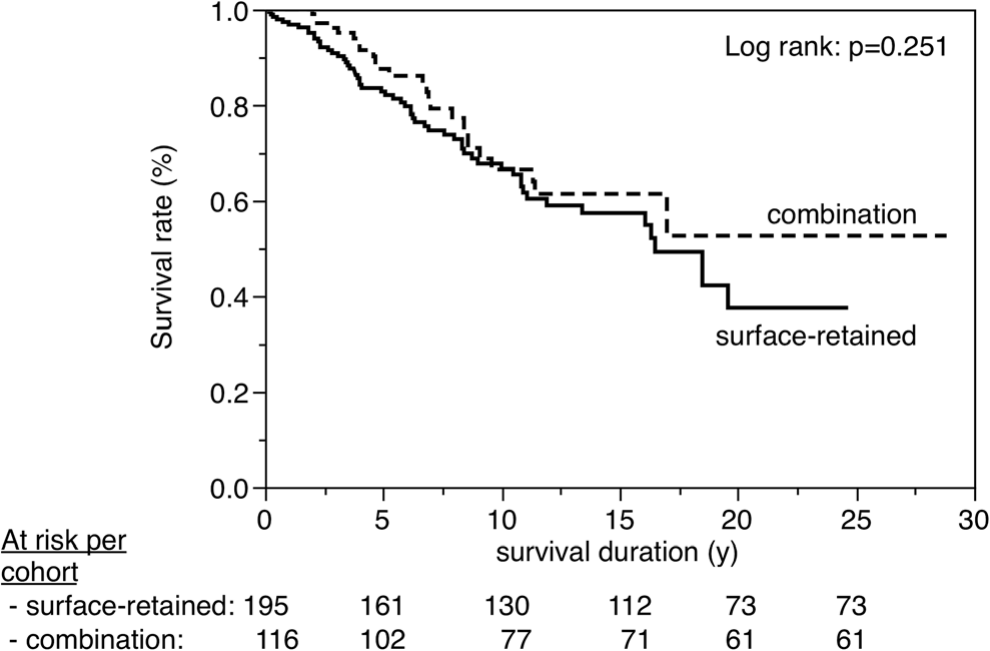
Survival in relation to framework structure (surface-retained versus combination)

**Fig. 9:**
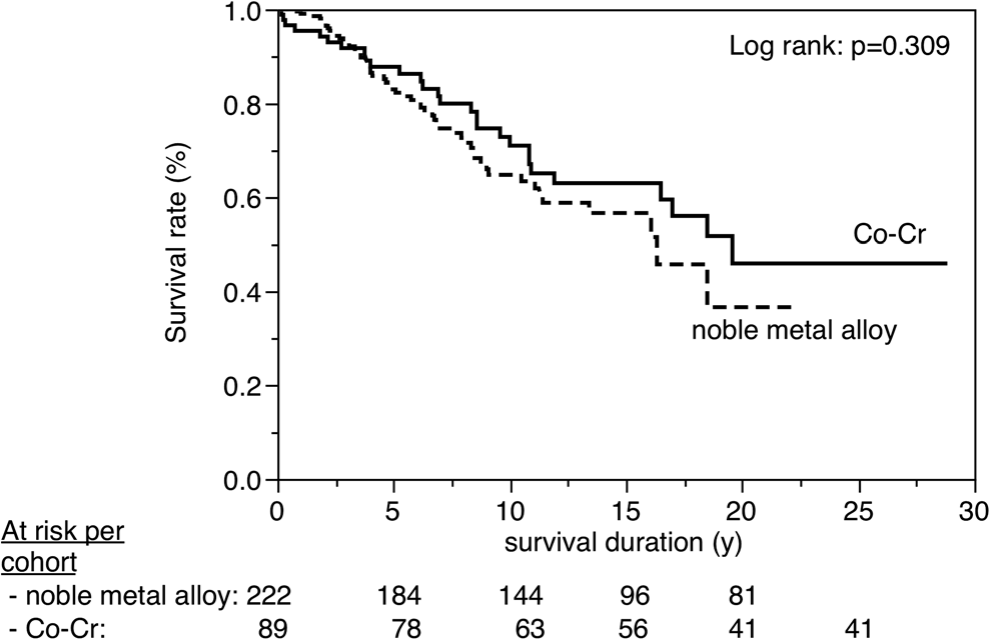
Survival in relation to framework alloy (noble metal alloys versus Co-Cr alloy)

**Fig. 10:**
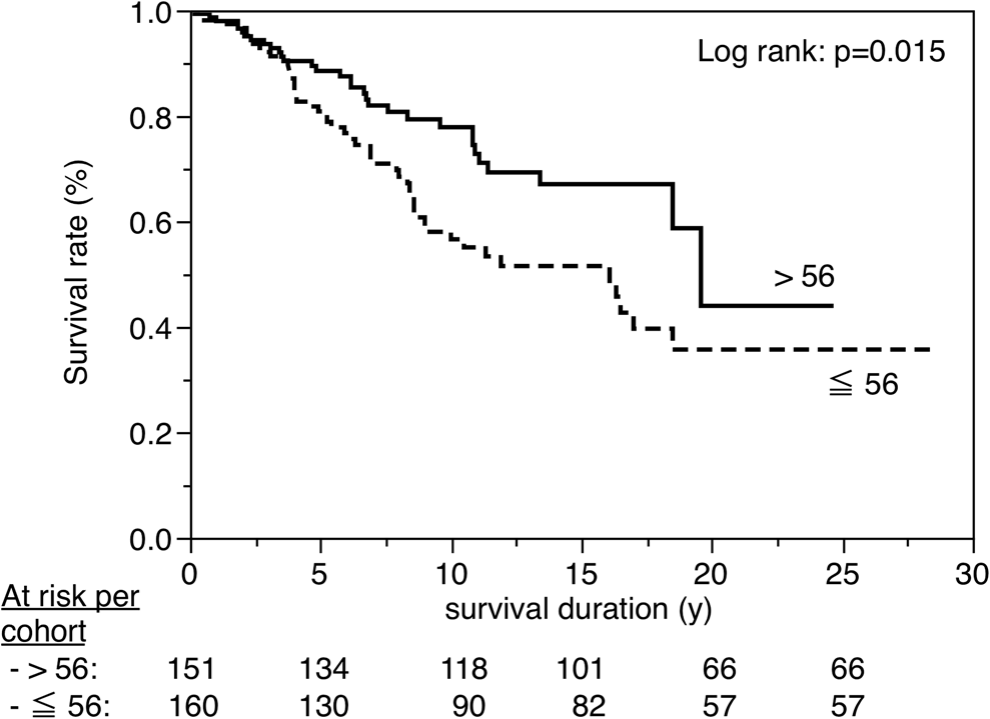
Survival in relation to age of the patient at insertion (≤56 versus >56)

**Fig. 11:**
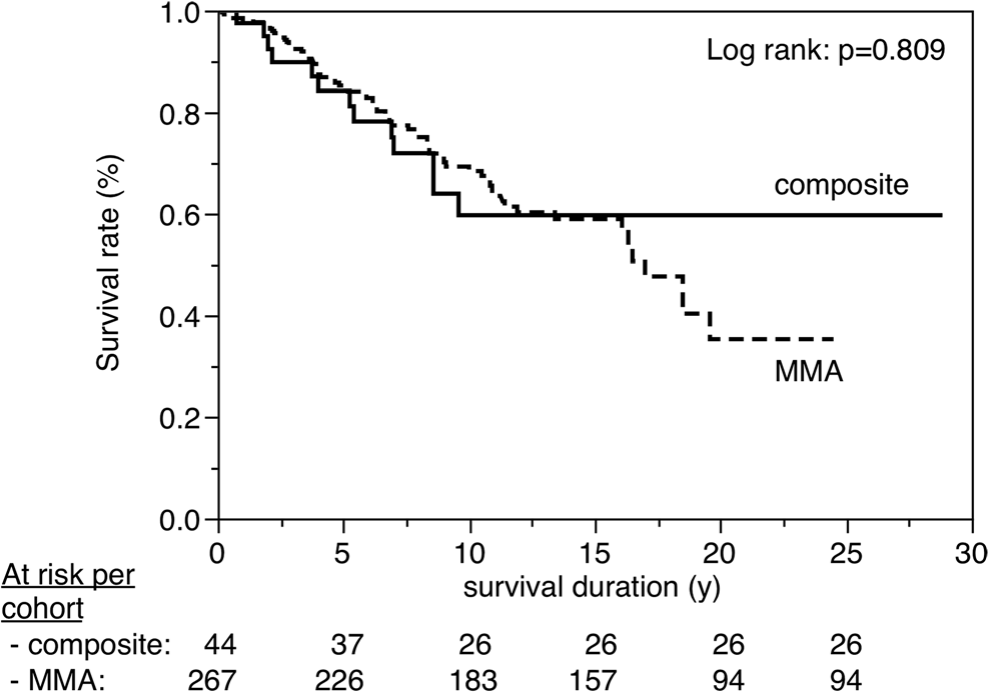
Survival in relation to cement type (MMA- versus composite-based)

**Fig. 12:**
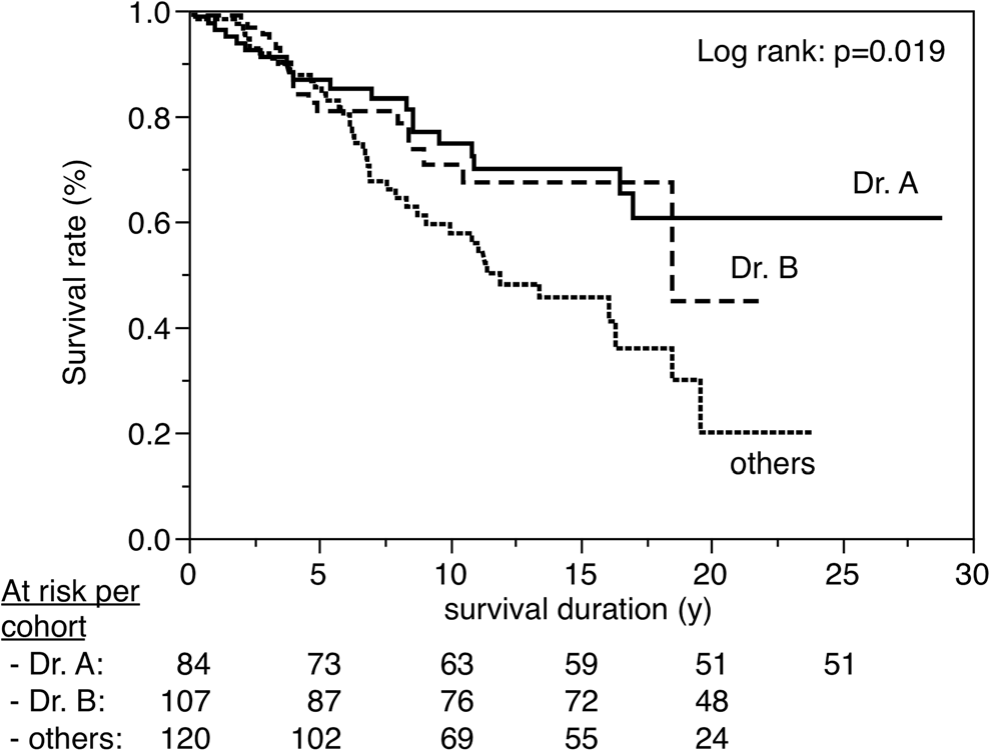
Survival in relation to difference in operator (Dr. A versus Dr. B versus others)

Figures 3, 4, 5, 6, 7, 8, 9, 10, 11, and 12 show the survival curves in relation to patient gender, location of the RBFPDP (maxilla versus mandible), location of the RBFPDP (anterior versus posterior), number of missing teeth, number of abutment teeth, framework structure, framework alloy, age of the patient at insertion, cement type, and different operators, respectively. Although the survival curves showed different tendencies, the variables had no statistical effect on the longevity after log-rank tests (*p* > 0.05), except for patient age (*p* = 0.015) and different operators (*p* = 0.019).

Considering the Kaplan–Meier analyses results, four factors were chosen as final models for the Cox proportional hazards regression analysis. The influence of covariables on survival ratios, including level of significance, hazard ratio, and 95 % confidence intervals, is shown in Table 2. Regarding the difference in operator variable, a statistical difference was indicated only between Dr. A and others.

**Table 2:** Influence of covariables on survival ratios, including level of significance, hazard ratio, and 95 % confidence intervals (final model)

Variable	Category	Number	Number of events	Hazard ratio (95 % confidence interval)	*P* value
Location	Maxilla^a^	181	55	0.679 (0.424–1.065)	0.093
	Mandible	130	29		
Number of abutment teeth	2^a^	133	64	0.716 (0.418–1.177)	0.192
	>2	78	20		
Age of patient	>56^a^	151	32	1.723 (1.112–2.710)	0.015
	≦56	160	52		
Operator	Dr. A^a^	84	20		
	Dr. B	107	20	1.147 (0.604–2.181)	0.673
	Others	120	44	2.023 (1.203–3.533)	0.001

^a^Reference category

## Discussion

A resin-bonded prosthesis is defined as a prosthesis that is luted to vital tooth structure, primarily enamel. A prosthesis that combines full coverage crown and surface retainers (combination design in this study) is therefore different from a RBFPDP with surface retainers only. However, Boemicke et al. compared the clinical performance of RBFPDPs with conventional and combined designs and reported that there was no significant difference between the 5-year cumulative survival rates of either prosthesis design [23]. Similarly in this study, as seen in Fig. 8, no significant difference was recognized between these two designs. Therefore, the combination design was also included in this study.

At the Nagasaki University Hospital, RBFPDPs have been applied clinically from the early 1980s. The follow-up period of about 30 years in this study is therefore sufficiently long, and the results should be characteristic when compared with reports with shorter observation periods.

For example, many researchers reported that maxillary RBFPDPs were more susceptible to failure than mandibular RBFPDPs [2, 13–17]. Creugers et al. reported that the anterior RBFPDP exhibited a significantly higher survival rate than posterior RBFPDPs [24], and Aggstaller et al. reported that the type of metal alloy material significantly affected the survival rate [19]. Nevertheless, these factors did not influence survival in this study, and some reports show similar results [9, 18–20]. Only two factors, i.e., patient age at insertion and difference in operator, statistically influenced the survival rate.

In this study, 18 dentists were classified into three groups: Dr. A, Dr. B, and others. Drs. A and B had performed RBFPDP treatment during the same period (25 years), but Dr. A started RBFPDP treatment with more experience than Dr. B. “Others” includes many dentists having less clinical experience. Therefore, when comparing by the experience of the dental practitioner, Dr. A was the most experienced, followed by Dr. B and others.

Gartnett et al. evaluated the survival of RBFPDPs provided for post-orthodontic hypodontia patients with missing maxillary lateral incisors and reported that senior members of staff achieved the highest survival rate and that other factors were unrelated to the results [25]. It is well known that the factor of operator skill has an influence on the prognosis of various dental treatments. Dobranszki et al. reported that orthodontic microscrew failure was statistically influenced by the operator factor [26], and Kim et al. evaluated the longevity of direct restorations and indicated that the student group showed significantly greater risk than the professor group [27]. Frankenberger et al. compared the influence of operator to that of material on the stability of resin composite for luting of ceramic inlays and clearly demonstrated the operator influence [28].

It is certain that other factors unevaluated in this study could affect the survival of RBFPDPs. The effectiveness of moisture control using rubber dam to increase the longevity of RBFPDPs has been indicated [11], and occlusal factors and parafunctional activity are also important in the success and failure of these restorations [15, 29]. Of course, the periodontal factor is significantly influential [17, 30]. As to the number of units, Pröbster and Henrich indicated that the multi-unit RBFPDP (more than four units) had a smaller probability of survival than three-unit restorations [9]. The retention value of RBFPDPs is reported to be directly affected by the preparation of the enamel substrate of the abutment tooth and by the thickness of the retainers [31, 32].

Consequently, the operator factor may largely include these factors, i.e., the “expert” may understand all other related factors. The reason why the overall survival rate of this study should be lower than our previous report [18] was that many inexperienced dentists were included for evaluation.

The results were also statistically influenced by patient age at the time of seating. Pröbster and Henrich reported that the age of the patient was not an influential factor, but this might be caused by the fact that the patient age was distributed at around 20 years [9]. Fracture of abutment tooth structure was observed in 10 cases in the older adult group in this study. These fractures were not counted as failures, because the frameworks were not damaged and bonding between the metal alloy and tooth substance was strongly maintained.

Tooth fracture may have occurred because of the fragility of tooth substance in older adults. Zheng et al. reported that the enamel surface became more prone to cracks with aging [33], and Bajaj et al. indicated that the fatigue crack growth resistance of human dentin decreases with both age of the tissue and dehydration [34]. One of the advantages of RBFPDPs is the ability to maintain the same occlusion as before preparation by minimal invasion. However, the physiological mobility of the abutment tooth should be limited by fixture with RBFPDPs. Especially in the case of RBFPDPs participating in mandibular lateral translation, the risk of tooth fracture may be greater.

Accidental tooth fracture was not observed in the younger group, because the tooth substance was more durable. For older adults at high risk of tooth fracture, a more classic prosthesis such as a metal onlay or 4/5 crown may be more effective at protecting the remaining tooth substance. The necessity for minimally invasive preparation may be valid according to the age of the patient, and the design of the prosthesis should differ according to the need to protect the tooth substance.

Debonding/loosening occurred in all generations, and debonded/loosened RBFPDPs could not always be rebonded even if they were surface-retained. Partial debonding could easily be missed at a recall or subsequent examination, as the debonding area could be imperceptible and the debonded/loosened RBFPDP may not detach. The results of this study still indicated the difficulty of reusing a RBFPDP, although there is in theory a greater possibility of rebonding an RBFPDP when compared with a FPD. Clinicians should pay special attention and try not to miss any barely perceptible debonding of an RBFPDP when undertaking oral hygiene treatment.
